# *catena*-Poly[[diaqua­dichlorido­manganese(II)]-μ-1,1′-bis­(1*H*-1,2,4-triazol-1-ylmeth­yl)ferrocene]

**DOI:** 10.1107/S1600536809053823

**Published:** 2009-12-24

**Authors:** Chong-Yu Shi, Xiao-Li Zhou, Bo Wu, Ni Zhu, Zhen-Shu Weia

**Affiliations:** a Editorial Department of Journal of Zhongzhou University, Zhongzhou University, Zhengzhou 450044, People’s Republic of China; b Experiment Administrative Center, Zhongzhou University, Zhengzhou 450044, People’s Republic of China; c Henan Association for Science and Technology, Zhengzhou 450003, People’s Republic of China

## Abstract

In the title complex, [FeMn(C_8_H_8_N_3_)_2_Cl_2_(H_2_O)_2_]_*n*_, the Mn^II^ atom, located on an inversion center, is octa­hedrally coordinated by two N atoms from two adjacent 1,1′-bis­(1*H*-1,2,4-triazol-1-ylmeth­yl)ferrocene (btmf) ligands and two Cl atoms forming the equatorial plane, with the axial positions occupied by two O atoms of coordinated water mol­ecules. The btmf ligands link adjoining Mn^II^ atoms into a zigzag chain along the *a* axis. The crystal structure is stabilized by inter­molecular O—H⋯N hydrogen bonds, which link the chains, forming a two-dimensional layer parallel to (10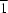); O—H⋯Cl inter­actions link the layers, forming a three-dimensional network.

## Related literature

For ferrocene complexes, see: Li *et al.* (2003 ▶); Beer (1992 ▶); Togni & Haltermann (1998 ▶); Gao *et al.* (2006 ▶); He *et al.* (2008 ▶). For related btmf complexes, see: Zhou *et al.* (2007 ▶); Sonoda & Moritani (1971 ▶); Wilkes *et al.* (1995 ▶).
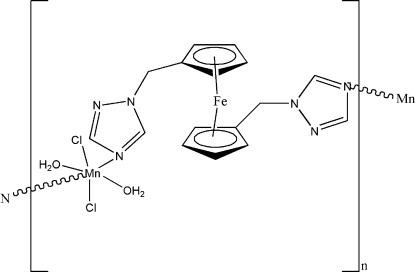


## Experimental

### 

#### Crystal data

[FeMn(C_8_H_8_N_3_)_2_Cl_2_(H_2_O)_2_]
*M* *_r_* = 510.07
Triclinic, 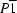

*a* = 5.9596 (7) Å
*b* = 7.1630 (8) Å
*c* = 12.4226 (14) Å
α = 98.963 (2)°
β = 101.076 (2)°
γ = 103.939 (2)°
*V* = 493.6 (1) Å^3^
*Z* = 1
Mo *K*α radiation
μ = 1.67 mm^−1^
*T* = 298 K
0.26 × 0.16 × 0.10 mm

#### Data collection

Bruker APEXII area-detector diffractometer
Absorption correction: multi-scan (*SADABS*; Bruker, 2005 ▶) *T*_min_ = 0.671, *T*_max_ = 0.851
2618 measured reflections
1751 independent reflections
1655 reflections with *I* > 2σ(*I*)
*R*_int_ = 0.007

#### Refinement

*R*[*F* ^2^ > 2σ(*F* ^2^)] = 0.027
*wR*(*F* ^2^) = 0.072
*S* = 1.06
1751 reflections
130 parameters
H-atom parameters constrained
Δρ_max_ = 1.32 e Å^−3^
Δρ_min_ = −0.32 e Å^−3^

### 

Data collection: *APEX2* (Bruker, 2005 ▶); cell refinement: *SAINT* (Bruker, 2005 ▶); data reduction: *SAINT*; program(s) used to solve structure: *SHELXS97* (Sheldrick, 2008 ▶); program(s) used to refine structure: *SHELXL97* (Sheldrick, 2008 ▶); molecular graphics: *ORTEPIII* (Burnett & Johnson, 1996 ▶) and *PLATON* (Spek, 2009 ▶); software used to prepare material for publication: *SHELXL97*.

## Supplementary Material

Crystal structure: contains datablocks I, global. DOI: 10.1107/S1600536809053823/dn2521sup1.cif

Structure factors: contains datablocks I. DOI: 10.1107/S1600536809053823/dn2521Isup2.hkl

Additional supplementary materials:  crystallographic information; 3D view; checkCIF report

## Figures and Tables

**Table 1:** Hydrogen-bond geometry (Å, °)

*D*—H⋯*A*	*D*—H	H⋯*A*	*D*⋯*A*	*D*—H⋯*A*
O1—H1*W*⋯Cl1^i^	0.83	2.28	3.1021 (17)	170
O1—H2*W*⋯N3^ii^	0.82	2.16	2.921 (3)	154

Symmetry codes: (i) 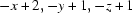; (ii) 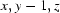.

**Fig. 1.**
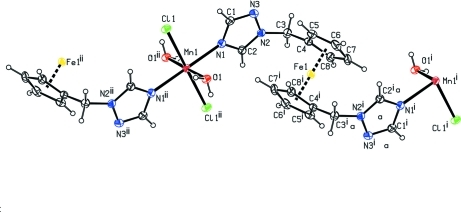
Molecular view of (I) with the atom-labeling scheme. Ellipsoids are drawn at the the 30% probability level. H atoms are shown as spheres of arbitrary radii. [Symmetric codes: (i) -x, 1-y, -z; (ii) -x+1, 1-y, 1-z].

**Fig. 2.**
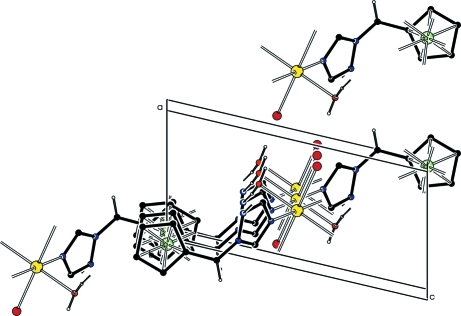
Partial packing view showing the formation of layers parallel to the (1 0 -1) plane through O-H···N hydrogen bonds. H atoms not involved in hydrogen bondings have been omitted for clarity.

## supplementary crystallographic information

### Comment

It is well known that ferrocene complexes undergo reversible redox reactions(Li *et al.*, 2003; Togni & Haltermann, 1998; Beer *et al.*, 1992). Moreover, ferrocene-based bidentate ligands are excellent building blocks and used to metal-organic polymers and supramolecular architectures(Gao *et al.*, 2006; He *et al.*, 2008). Recently, we have been employed ferrocene-containing ligand 1,1'-bis[(1H-1,2,4-triazol-1- yl)methyl]ferrocene(btmf) to construct Co(II) metal-organic polymer (Zhou *et al.*, 2007). Following on from our research work on the coordination chemistry of ferrocene-based bidentate, we focused on the effect of metal ions on the metal-organic polymers. In this paper, we report here the synthesis and crystal structure of the title complex (I).

The Mn^II^ and iron atoms are located on inversion center. Mn^II^ is octahedrally coordinated by two N atoms from two adjacent btmf ligands and two Cl atoms forming the equatorial plane, whereas axial positions are occupied by two O atoms of coordinated water molecules. The distances of Mn-N, Mn-Cl and Mn-O bonds are within normal range. The torsion angle between ferrocene moiety and triazole motif is 62.4 (3)°, which is near to our reported Co^II^ compound(Zhou *et al.*, 2007). Each btmf molecular clips serve as bridge to link two adjacent Mn^II^ centers into a 1D zigzag chain, as shown in Fig. 1. Comparison of {Co(btmf)_2_(CH_3_CH_2_OH)(H_2_O)] ClO_4_)_2_.3(CH_3_CH_2_OH)}_n_ (Zhou *et al.*, 2007) and the title compound indicates that the metal ions may play critical role in modulating the resulting structure.

The structure is further stabilized by intermolecular O-H···N, O-H···Cl hydrogen bonds(Table. 1). The O-H···N hydrogen bonds links the chains forming a two dimensionnal layer parallel to the (1 0 -1) plane (Fig. 2) whereas the O-H···Cl interactions links these layers to form a thre dimensionnal network.

### Experimental

The preparation of btmf ligand followed established method presented in the literature (Wilkes *et al.*, 1995, Sonoda *et al.*, 1971). The synthesis process of the title compound of (I) was same as the {Co(btmf)_2_(CH_3_CH_2_OH)(H_2_O)] ClO_4_)_2_.3(CH_3_CH_2_OH)}^n^ (Zhou *et al.*, 2007) compound except for Co(ClO_4_)_2_ by MnCl_2_(1.3mg, 0.01mmol). The well colorless crystals were obtained.

### Refinement

The H atoms of btmf ligand were placed in geometrically idealized positions and constrained to ride on their parent atoms, with C-H distances in the range 0.93-0.98Å, with U_iso_(H)=1.2U_eq_(C). The H atoms of water molecules were located in a difference map and refined with restraints of O-H=0.83 (1)Å, and with U_iso_(H)=1.5U_eq_(O). The highest peak of residual density is located 1.94Å from C4 atom.

### Crystal data

**Table:**

[FeMn(C_8_H_8_N_3_)_2_Cl_2_(H_2_O)_2_]	*Z* = 1
*M**_r_* = 510.07	*F*(000) = 259
Triclinic, *P*1	*D*_x_ = 1.716 Mg m^−^^3^
Hall symbol: -P 1	Mo *K*α radiation, λ = 0.71073 Å
*a* = 5.9596 (7) Å	Cell parameters from 1751 reflections
*b* = 7.1630 (8) Å	θ = 3.0–25.2°
*c* = 12.4226 (14) Å	µ = 1.67 mm^−^^1^
α = 98.963 (2)°	*T* = 298 K
β = 101.076 (2)°	Block, colorless
γ = 103.939 (2)°	0.26 × 0.16 × 0.10 mm
*V* = 493.6 (1) Å^3^	

### Data collection

**Table:**

Bruker APEXII area-detector diffractometer	1751 independent reflections
Radiation source: fine-focus sealed tube	1655 reflections with *I* > 2σ(*I*)
graphite	*R*_int_ = 0.007
φ and ω scan	θ_max_ = 25.2°, θ_min_ = 3.0°
Absorption correction: multi-scan (*SADABS*; Bruker, 2005)	*h* = −6→7
*T*_min_ = 0.671, *T*_max_ = 0.851	*k* = −8→8
2618 measured reflections	*l* = −13→14

### Refinement

**Table:**

Refinement on *F*^2^	Primary atom site location: structure-invariant direct methods
Least-squares matrix: full	Secondary atom site location: difference Fourier map
*R*[*F*^2^ > 2σ(*F*^2^)] = 0.027	Hydrogen site location: inferred from neighbouring sites
*wR*(*F*^2^) = 0.072	H-atom parameters constrained
*S* = 1.06	*w* = 1/[σ^2^(*F*_o_^2^) + (0.035*P*)^2^ + 0.4359*P*] where *P* = (*F*_o_^2^ + 2*F*_c_^2^)/3
1751 reflections	(Δ/σ)_max_ = 0.001
130 parameters	Δρ_max_ = 1.32 e Å^−^^3^
0 restraints	Δρ_min_ = −0.32 e Å^−^^3^

### Special details

**Table:**

Geometry. All esds (except the esd in the dihedral angle between two l.s. planes) are estimated using the full covariance matrix. The cell esds are taken into account individually in the estimation of esds in distances, angles and torsion angles; correlations between esds in cell parameters are only used when they are defined by crystal symmetry. An approximate (isotropic) treatment of cell esds is used for estimating esds involving l.s. planes.
Refinement. Refinement of F^2^ against ALL reflections. The weighted R-factor wR and goodness of fit S are based on F^2^, conventional R-factors R are based on F, with F set to zero for negative F^2^. The threshold expression of F^2^ > 2sigma(F^2^) is used only for calculating R-factors(gt) etc. and is not relevant to the choice of reflections for refinement. R-factors based on F^2^ are statistically about twice as large as those based on F, and R- factors based on ALL data will be even larger.

### Fractional atomic coordinates and isotropic or equivalent isotropic displacement parameters (Å^2^)

**Table:**

	*x*	*y*	*z*	*U*_iso_*/*U*_eq_	
Fe1	0.0000	0.5000	0.0000	0.02935 (14)	
Mn1	0.5000	0.5000	0.5000	0.02631 (14)	
Cl1	0.90203 (9)	0.74119 (8)	0.57695 (5)	0.03530 (16)	
O1	0.6247 (3)	0.3669 (2)	0.35390 (13)	0.0326 (4)	
H1W	0.7574	0.3523	0.3780	0.049*	
H2W	0.5284	0.2583	0.3270	0.049*	
N1	0.3881 (3)	0.7148 (3)	0.40001 (16)	0.0314 (4)	
N2	0.1856 (3)	0.8317 (3)	0.27483 (15)	0.0290 (4)	
N3	0.4163 (4)	0.9406 (3)	0.29279 (18)	0.0372 (5)	
C1	0.5298 (4)	0.8646 (3)	0.3686 (2)	0.0343 (5)	
H1	0.6935	0.9100	0.3982	0.041*	
C2	0.1739 (4)	0.7009 (3)	0.33907 (19)	0.0316 (5)	
H2	0.0340	0.6112	0.3413	0.038*	
C3	−0.0041 (4)	0.8607 (4)	0.19119 (19)	0.0341 (5)	
H3A	0.0119	1.0005	0.1998	0.041*	
H3B	−0.1564	0.7989	0.2052	0.041*	
C4	−0.0033 (4)	0.7779 (3)	0.07253 (19)	0.0291 (5)	
C5	0.1944 (5)	0.7898 (3)	0.0235 (2)	0.0374 (6)	
H5	0.3535	0.8445	0.0611	0.045*	
C6	0.1073 (6)	0.7037 (4)	−0.0928 (2)	0.0472 (7)	
H6	0.1995	0.6909	−0.1446	0.057*	
C7	−0.1441 (6)	0.6408 (4)	−0.1162 (2)	0.0477 (7)	
H7	−0.2464	0.5801	−0.1860	0.057*	
C8	−0.2126 (5)	0.6868 (4)	−0.0143 (2)	0.0374 (6)	
H8	−0.3681	0.6615	−0.0058	0.045*	

### Atomic displacement parameters (Å^2^)

**Table:**

	*U*^11^	*U*^22^	*U*^33^	*U*^12^	*U*^13^	*U*^23^
Fe1	0.0377 (3)	0.0268 (2)	0.0252 (2)	0.0149 (2)	0.00395 (19)	0.00533 (18)
Mn1	0.0237 (3)	0.0282 (3)	0.0254 (3)	0.00630 (19)	0.00377 (18)	0.00518 (19)
Cl1	0.0235 (3)	0.0334 (3)	0.0426 (3)	0.0039 (2)	0.0030 (2)	0.0017 (2)
O1	0.0270 (8)	0.0310 (8)	0.0367 (9)	0.0056 (7)	0.0068 (7)	0.0032 (7)
N1	0.0306 (10)	0.0324 (10)	0.0294 (10)	0.0076 (8)	0.0049 (8)	0.0060 (8)
N2	0.0316 (10)	0.0268 (9)	0.0271 (10)	0.0090 (8)	0.0051 (8)	0.0026 (7)
N3	0.0344 (11)	0.0319 (11)	0.0405 (12)	0.0027 (9)	0.0042 (9)	0.0098 (9)
C1	0.0287 (12)	0.0330 (12)	0.0362 (13)	0.0044 (10)	0.0023 (10)	0.0058 (10)
C2	0.0309 (12)	0.0330 (12)	0.0306 (12)	0.0077 (10)	0.0080 (9)	0.0073 (9)
C3	0.0369 (13)	0.0349 (13)	0.0318 (12)	0.0183 (10)	0.0042 (10)	0.0025 (10)
C4	0.0353 (12)	0.0233 (11)	0.0312 (12)	0.0138 (9)	0.0057 (9)	0.0069 (9)
C5	0.0447 (15)	0.0290 (12)	0.0425 (14)	0.0118 (11)	0.0153 (11)	0.0109 (10)
C6	0.076 (2)	0.0427 (15)	0.0377 (14)	0.0286 (14)	0.0258 (14)	0.0191 (12)
C7	0.076 (2)	0.0406 (14)	0.0286 (13)	0.0322 (14)	−0.0025 (12)	0.0076 (11)
C8	0.0408 (14)	0.0335 (12)	0.0382 (13)	0.0204 (11)	−0.0008 (11)	0.0055 (10)

### Geometric parameters (Å, °)

**Table:**

Fe1—C6	2.056 (3)	N1—C1	1.356 (3)
Fe1—C6^i^	2.056 (3)	N2—C2	1.320 (3)
Fe1—C7^i^	2.057 (2)	N2—N3	1.364 (3)
Fe1—C7	2.057 (2)	N2—C3	1.460 (3)
Fe1—C4	2.060 (2)	N3—C1	1.317 (3)
Fe1—C4^i^	2.060 (2)	C1—H1	0.9300
Fe1—C8^i^	2.057 (2)	C2—H2	0.9300
Fe1—C8	2.057 (2)	C3—C4	1.502 (3)
Fe1—C5	2.064 (2)	C3—H3A	0.9700
Fe1—C5^i^	2.064 (2)	C3—H3B	0.9700
Mn1—O1^ii^	2.2527 (16)	C4—C5	1.417 (3)
Mn1—O1	2.2527 (16)	C4—C8	1.420 (3)
Mn1—N1^ii^	2.2639 (19)	C5—C6	1.421 (4)
Mn1—N1	2.2639 (19)	C5—H5	0.9300
Mn1—Cl1	2.5000 (6)	C6—C7	1.414 (4)
Mn1—Cl1^ii^	2.5000 (6)	C6—H6	0.9300
O1—H1W	0.8274	C7—C8	1.418 (4)
O1—H2W	0.8224	C7—H7	0.9300
N1—C2	1.326 (3)	C8—H8	0.9300
			
C6—Fe1—C6^i^	180.00 (11)	O1—Mn1—Cl1^ii^	89.68 (4)
C6—Fe1—C7^i^	139.78 (13)	N1^ii^—Mn1—Cl1^ii^	89.38 (5)
C6^i^—Fe1—C7^i^	40.22 (13)	N1—Mn1—Cl1^ii^	90.62 (5)
C6—Fe1—C7	40.22 (13)	Cl1—Mn1—Cl1^ii^	180.0
C6^i^—Fe1—C7	139.78 (13)	Mn1—O1—H1W	108.5
C7^i^—Fe1—C7	180.00 (11)	Mn1—O1—H2W	105.9
C6—Fe1—C4	67.93 (10)	H1W—O1—H2W	109.2
C6^i^—Fe1—C4	112.07 (10)	C2—N1—C1	102.58 (19)
C7^i^—Fe1—C4	112.05 (10)	C2—N1—Mn1	128.23 (16)
C7—Fe1—C4	67.95 (10)	C1—N1—Mn1	127.89 (16)
C6—Fe1—C4^i^	112.07 (10)	C2—N2—N3	109.41 (19)
C6^i^—Fe1—C4^i^	67.93 (10)	C2—N2—C3	129.1 (2)
C7^i^—Fe1—C4^i^	67.95 (10)	N3—N2—C3	121.46 (19)
C7—Fe1—C4^i^	112.05 (10)	C1—N3—N2	102.70 (19)
C4—Fe1—C4^i^	180.0	N3—C1—N1	114.4 (2)
C6—Fe1—C8^i^	112.35 (11)	N3—C1—H1	122.8
C6^i^—Fe1—C8^i^	67.65 (11)	N1—C1—H1	122.8
C7^i^—Fe1—C8^i^	40.31 (11)	N2—C2—N1	110.9 (2)
C7—Fe1—C8^i^	139.69 (11)	N2—C2—H2	124.5
C4—Fe1—C8^i^	139.64 (9)	N1—C2—H2	124.5
C4^i^—Fe1—C8^i^	40.36 (9)	N2—C3—C4	113.50 (18)
C6—Fe1—C8	67.65 (11)	N2—C3—H3A	108.9
C6^i^—Fe1—C8	112.35 (11)	C4—C3—H3A	108.9
C7^i^—Fe1—C8	139.69 (11)	N2—C3—H3B	108.9
C7—Fe1—C8	40.31 (11)	C4—C3—H3B	108.9
C4—Fe1—C8	40.36 (9)	H3A—C3—H3B	107.7
C4^i^—Fe1—C8	139.64 (9)	C5—C4—C8	107.6 (2)
C8^i^—Fe1—C8	180.0	C5—C4—C3	128.2 (2)
C6—Fe1—C5	40.36 (11)	C8—C4—C3	124.0 (2)
C6^i^—Fe1—C5	139.64 (11)	C5—C4—Fe1	70.05 (13)
C7^i^—Fe1—C5	112.36 (11)	C8—C4—Fe1	69.72 (12)
C7—Fe1—C5	67.64 (11)	C3—C4—Fe1	129.76 (16)
C4—Fe1—C5	40.21 (9)	C4—C5—C6	108.2 (2)
C4^i^—Fe1—C5	139.79 (9)	C4—C5—Fe1	69.75 (13)
C8^i^—Fe1—C5	112.51 (10)	C6—C5—Fe1	69.51 (15)
C8—Fe1—C5	67.49 (10)	C4—C5—H5	125.9
C6—Fe1—C5^i^	139.64 (11)	C6—C5—H5	125.9
C6^i^—Fe1—C5^i^	40.36 (11)	Fe1—C5—H5	126.4
C7^i^—Fe1—C5^i^	67.64 (11)	C7—C6—C5	108.0 (2)
C7—Fe1—C5^i^	112.36 (11)	C7—C6—Fe1	69.95 (15)
C4—Fe1—C5^i^	139.79 (9)	C5—C6—Fe1	70.13 (14)
C4^i^—Fe1—C5^i^	40.21 (9)	C7—C6—H6	126.0
C8^i^—Fe1—C5^i^	67.49 (10)	C5—C6—H6	126.0
C8—Fe1—C5^i^	112.51 (10)	Fe1—C6—H6	125.5
C5—Fe1—C5^i^	180.0	C6—C7—C8	107.9 (2)
O1^ii^—Mn1—O1	180.00 (5)	C6—C7—Fe1	69.83 (14)
O1^ii^—Mn1—N1^ii^	89.33 (6)	C8—C7—Fe1	69.84 (13)
O1—Mn1—N1^ii^	90.67 (6)	C6—C7—H7	126.0
O1^ii^—Mn1—N1	90.67 (6)	C8—C7—H7	126.0
O1—Mn1—N1	89.33 (6)	Fe1—C7—H7	125.9
N1^ii^—Mn1—N1	180.00 (8)	C4—C8—C7	108.3 (2)
O1^ii^—Mn1—Cl1	89.68 (4)	C4—C8—Fe1	69.93 (12)
O1—Mn1—Cl1	90.32 (4)	C7—C8—Fe1	69.85 (14)
N1^ii^—Mn1—Cl1	90.62 (5)	C4—C8—H8	125.8
N1—Mn1—Cl1	89.38 (5)	C7—C8—H8	125.8
O1^ii^—Mn1—Cl1^ii^	90.32 (4)	Fe1—C8—H8	126.0

Symmetry codes: (i) −*x*, −*y*+1, −*z*; (ii) −*x*+1, −*y*+1, −*z*+1.

### Hydrogen-bond geometry (Å, °)

**Table:**

*D*—H···*A*	*D*—H	H···*A*	*D*···*A*	*D*—H···*A*
O1—H1W···Cl1^iii^	0.83	2.28	3.1021 (17)	170
O1—H2W···N3^iv^	0.82	2.16	2.921 (3)	154

Symmetry codes: (iii) −*x*+2, −*y*+1, −*z*+1; (iv) *x*, *y*−1, *z*.
